# Alfred Whitmore and the Discovery of Melioidosis

**DOI:** 10.3201/eid3004.230693

**Published:** 2024-04

**Authors:** Jelmer Savelkoel, David A.B. Dance

**Affiliations:** Amsterdam UMC location University of Amsterdam, Amsterdam, the Netherlands (J. Savelkoel);; Lao-Oxford-Mahosot Hospital–Wellcome Trust Research Unit, Mahosot Hospital, Vientiane, Laos (D.A.B. Dance);; University of Oxford, Oxford, UK (D.A.B. Dance);; London School of Hygiene & Tropical Medicine, London, UK (D.A.B. Dance)

**Keywords:** Melioidosis, Burkholderia pseudomallei, bacteria, history

## Abstract

We review the discovery of the tropical infectious disease melioidosis by Alfred Whitmore, a pathologist from England, and his assistant from India, C.S. Krishnaswami. We discuss how the subsequent disappearance of melioidosis from the medical literature of Burma holds parallels with the current neglect and under recognition of the disease. We urge global and national public health authorities to add melioidosis to existing neglected tropical diseases surveillance systems.

“We have, and always will have, need of both Science and Art in medical practice; they are not antagonistic principles, but are mutually helpful; there is room, enough and to spare, for the free and energetic use of both; it is ignorance alone which sees them hostile, folly indeed which seeks their division.” So wrote Alfred Whitmore in 1914 (*1*). Whitmore was a remarkable man, not only in advocating what seems like a modern integration between laboratory and clinical work more than 100 years ago (*1*) but also in achieving eponymous immortality by describing a novel disease (*2*). He did this without specialist training and while working in relative isolation in his role as pathologist in Rangoon General Hospital (RGH), Burma (now Myanmar), in 1911 (*2*,*3*). Yet the way in which his findings were initially fêted and subsequently forgotten holds strong parallels with the present day under recognition and ignorance of both Whitmore and the disease he described.

The disease discovered by Whitmore and his assistant, C.S. Krishnaswami, is now known as melioidosis but is still often referred to as Whitmore’s disease (*2*,*4*). The name melioidosis was only later coined by A.T. Stanton and W. Fletcher in 1921 and is derived from a Greek word meaning glanders-like disease (*5*,*6*). Over the past few years, and captivated by Whitmore’s wonderful prose style, one of us has been researching his life and work (*7*–*9*). In this review, and the accompanying video (Figure 3), we summarize some of our more recent findings about Whitmore’s career. We discuss what happened to his discovery in the years after he made it, and how that relates to melioidosis today. The information included in this review is based on the references provided and documents kindly provided by the Whitmore family.

**Figure 3:**
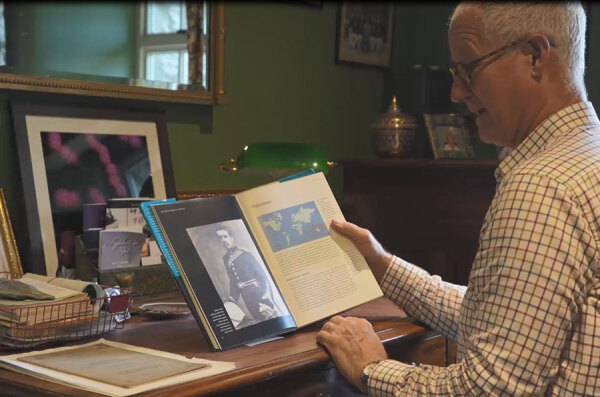
David Dance describing his research journey into the life and times of Alfred Whitmore and the discovery of melioidosis. Dance’s research included close collaboration with the Whitmore family and visits to archives in India, Myanmar, and the United Kingdom. (Video link: https://vimeo.com/925317363?share=copy)

Whitmore was born in Botcherby, in northwest England, in 1876 and grew up in Sebergham in Cumbria, where his father was rector (*10*). Among his early hobbies, Whitmore studied the process of decay of animal carcasses, which might have presaged his later career. He attended St. Bees School in Cumbria, studied medicine at Gonville and Caius College at Cambridge University, and completed his medical training at St. Mary’s Hospital in London, receiving scholarships and prizes along the way (*10*). After qualifying as a doctor, he completed the Diploma in Public Health that was obligatory for entrants into the Indian Medical Service (IMS) and was commissioned as a lieutenant in January 1903 (Figure 1) (*10*,*11*). His posting to the less fashionable center of Burma might have been influenced by his finishing last in the Diploma in Public Health exam. In later life he commented, “I entered the Service with no particular ambition, chiefly perhaps because I had no money—a bad reason.” His mandatory 2 years of military service were spent in India and the Andaman Islands before he arrived in Rangoon, Burma, toward the end of 1905 (*10*), little expecting that he would shortly unearth a novel infectious disease.

**Figure 1:**
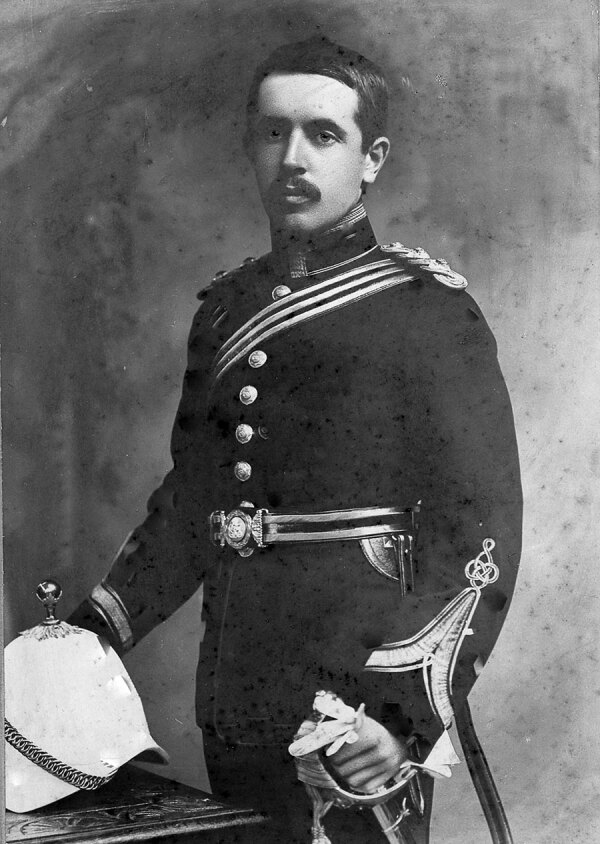
Alfred Whitmore wearing his Indian Medical Service uniform, circa 1903.

In the old, wooden RGH, Whitmore’s initial responsibility was taking care of the dying patients on the “moribund ward,” without the aid of nurses and with hygiene that left much to be desired. He even mentioned rumors of patients’ feet being gnawed off by rats in a letter written to his son in January 1946, although adding that he could not “aver the truth of this.” He thought the care that was provided was undignified for any human being. Thus, he tried to improve the lot of his patients by providing alcohol, which he described as the “best med of any that I have used,” as well as cheroots (a local type of cigar) and a wheeled bath that he later found being used as a fish tank. However, his role was about to change because plans were underway to build a new hospital, albeit without a laboratory (*12*). That plan was contrary to Alfred’s ideas. In a talk given in Cambridge following his return to England, he said he was “… trained to believe that there was a ‘Science’ as well as an ‘Art’ of Medicine and that the laboratory was the High Altar, as it were, of that Science.” He convinced the authorities of the necessity of a laboratory, which was duly added, with Whitmore taking on the role of pathologist on condition that he would also undertake the medicolegal work in the capacity of police surgeon. He remained a passionate advocate for the integration of a laboratory within the hospital, believing that close collaboration between the clinic and laboratory were essential to good quality “Western medicine” (*1*).

Whitmore worked as pathologist and police surgeon at RGH from 1909 until 1915, during which he discovered melioidosis (*10*). Melioidosis, caused by the gram-negative bacterium *Burkholderia pseudomallei*, is now known as a disease that typically has clinical manifestations of pneumonia, sepsis, and abscess formation, predominantly in persons with underlying risk factors, details of which can be found elsewhere (*13*). 

In 1911, Whitmore and Krishnaswami, who had graduated as a licentiate in medicine and surgery from Madras Medical College (*14*), undertook a postmortem examination that revealed “…a peculiar consolidation of the lungs,” which they felt was consistent with glanders, a zoonotic disease of horses (*2*). The victim, however, had no history of recent animal exposure, and cultures yielded a bacterium that they recognized as different from what was then known as *Bacillus mallei *(now *Burkholderia mallei*), the cause of glanders, in its rapid luxuriant growth and motility. In 1912, they published, in the Indian Medical Gazette, their microbiological and pathological findings from 38 cases of this recently discovered disease (*2*). A more comprehensive report in the subsequent year, in which Whitmore first proposed the specific epithet pseudomallei, included additional information about each case, 31 of which were “morphine injectors” (*15*). By 1914 the term morphia injectors’ septicaemia had been suggested for the disease name (*16*). 

Opioid injection is not currently recognized as a risk factor for melioidosis, and the reasons for such a strong association as that reported by Whitmore are unclear (*13*). Whitmore himself initially felt the infection most likely was caused by the general debility associated with morphine injections (*15*) but later appears to have favored contamination of the injections themselves, because 46 of the 52 cases in the 1914 report also bore evidence of morphine injection (*16*). That finding would hardly have been surprising given the squalid conditions in which the drug users of the day were liable to have received their morphine (*17*). 

Then along came World War I, and as a member of the IMS, Whitmore was obliged to return to military service in British India (*10*). Meanwhile, Krishnaswami continued to work on the disease until 1917, when he stated he had encountered some 200 cases (*18*), but he subsequently moved to work in what was then known as the “Lunatic Asylum” system for the rest of his career in Burma (*14*,*19*).

Whitmore spent the war at various stations in British India (*10*), frequently having brushes with authority because he did not tolerate fools, especially if they were senior officers issuing edicts from “the hill tops.” On returning to Rangoon, although he had hoped to resume his job as pathologist, he was appointed as a civil surgeon and never worked on melioidosis again (*10*). He found his role as a civil surgeon exhausting, leaving him little time for the reading he considered essential to keep up to date with the latest developments. In 1922, he moved to become superintendent of the Burma Government Medical School, a role in which he remained until he returned to England on leave in 1924, eventually retiring from the IMS in 1927 (*10*,*20*–*22*). 

After a brief spell in Paignton, Devon, UK, Whitmore moved to Madingley, near Cambridge, UK, where he remained involved in education and research until his death in 1946 (Figure 2) (*23*). People thought of him as an inspirational lecturer, and his obituary in the British Medical Journal described him as “… a most lovable man with a very keen sense of humour, and all who knew him well were greatly attached to him. He seemed to radiate something buoyant and joyous, and the outlook always seemed brighter when he was about” (*23*).

**Figure 2:**
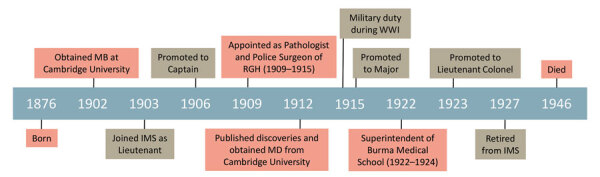
Timeline of the life of Alfred Whitmore and the discovery of melioidosis. The timeline highlights Whitmore’s personal and military achievements. IMS, Indian Medical Service; MB, bachelor of medicine degree; RGH, Rangoon General Hospital; WWI, World War I.

Whitmore’s discovery was initially celebrated by the colonial hierarchy in Burma and was specifically mentioned in the Report on the Administration of Burma for 2 consecutive years (1911–12 and 1912–13) (*24*,*25*). Lengthy debates about the appropriate form of bacteriologic support for the government in Burma had taken place, and some suggested that locating a laboratory in Rangoon would be impossible because of the climate; therefore, the authorities were doubtless keen to celebrate the success of this innovative venture (*12*,*26*). Yet, when World War I intervened, and Whitmore and Krishnaswami subsequently turned their attention to other duties, no one appears to have followed up on their findings: the next mention of melioidosis in Burma was in the 1940s (*27*). At that time, the disease appeared almost exclusively to affect the “friendless wastrels” of Rangoon, primarily persons addicted to morphine, who were unlikely ever to be economically productive and who, through crime, might be a drain on colonial resources (*15*,*28*). In addition, up to that point, not a single case of melioidosis had been reported in a person of European heritage. Those factors must undoubtedly have been key in the apparent neglect of the disease by the colonial authorities as they struggled to restore order after the disruption of World War I.

Today, melioidosis remains a little-known disease, even within the countries where it is endemic (*29*). Melioidosis endemic regions include northern Australia, many countries in South and Southeast Asia, sub-Saharan Africa, and tropical and subtropical areas of the Americas (*30*,*31*). Melioidosis is not yet formally recognized by the World Health Organization as a neglected tropical disease (NTD), despite the growing evidence that it is widespread throughout the tropics, is estimated to cause nearly 90,000 deaths a year globally, and has a disease burden considerably greater than that of many officially recognized NTDs (*31*–*33*). However, as a disease that mainly affects lower income populations living in rural areas, another marginalized group, melioidosis remains underdiagnosed and excluded from the mandatory surveillance systems of most countries where it is endemic. A 2021 multistate outbreak in the United States from an imported aromatherapy spray and a 2022 report of isolation of *B. pseudomallei* from the environment in Mississippi, USA, for the first time, might have temporarily boosted the profile of melioidosis (*34*,*35*). Perhaps the increased attention resulting from those incidents will benefit the communities that suffer from this deadly disease. 

In conclusion, global and national public health authorities should act to add melioidosis to NTD surveillance systems so that we do not again fail to follow the path initially signposted by Whitmore. We need to prevent history from repeating itself and make sure that both melioidosis, and Alfred Whitmore, are no longer forgotten.
